# Is Environmental Pollution Associated with an Increased Number of COVID-19 Cases in Europe?

**DOI:** 10.3390/ijerph19020703

**Published:** 2022-01-08

**Authors:** Javier Cifuentes-Faura

**Affiliations:** Department of Financial Economics and Accounting, Faculty of Economics and Business, University of Murcia, 30100 Murcia, Spain; javier.cifuentes@um.es

**Keywords:** COVID-19, pollutant gases, carbon monoxide, pollutant emissions from transport, sustainability, Europe

## Abstract

COVID-19 has caused an unprecedented crisis, resulting in a global pandemic with millions infected and dying. Given the importance given to sustainability and the reduction in pollutant gases in recent years, the main objective of this study was to determine whether pollutant emissions are associated with an increased number of COVID-19 cases in Europe. Other demographic variables that may have an impact on the number of coronavirus cases, such as population density, average age or the level of restrictive policies implemented by governments, are also included. It has been shown that the emission of carbon monoxide pollutant gases and pollutant emissions from transport positively affect the incidence of COVID-19, so that the sustainable policy implemented in recent years in Europe should be reinforced, and tougher sanctions and measures should be imposed when pollution thresholds are exceeded.

## 1. Introduction

The emergence of COVID-19 and its rapid spread has been an unprecedented crisis, affecting all areas, from health [1,2] to economic [3,4] and social [5,6] perspectives. The European continent has been one of the most affected in terms of number of cases and deaths. To curb the spread of the pandemic, exceptional measures were taken such as the perimeter confinement of many cities and mobility restrictions [7,8,9]. The pandemic has also led to the paralysis of many important sectors of activity, such as tourism and catering, which were major contributors to gross domestic product and economic growth [10,11].

With the development of the first vaccines to combat the disease, there has been a breakthrough in the fight against the pandemic [12]; however, there are still many sectors and people affected by the disease. During the pandemic, whether environmental pollution could also lead to an increase in the number of coronavirus cases has been questioned [13,14]. In addition, population density has been considered to be related to coronavirus cases [15], as well as the average age of the population or the level of policies implemented by governments.

In this paper we aim to measure the association that environmental variables, such as carbon gas emissions or pollutant gases from transport, may have with coronavirus cases. We also use other variables that the scientific literature considers as having a possible effect on the number of cases, such as the population density of European Union countries, the strict policies employed by countries to contain the pandemic and the average age of the population. To date, there has been no published work to date that combines these environmental variables for the European continent. In addition to the interest and need to know more about the coronavirus pandemic, this work may provide new information for policy makers.

Some work also shows that air pollution can contribute to a higher rate of COVID-19 infection and mortality [14]. This was demonstrated by some investigations for regions in China [13,16], where a positive correlation was observed between COVID-19 cases and air pollution indicators [17]. Fattorini and Regoli [18] and Frontera et al. [19] also showed a positive correlation in Italy, where more COVID-19 cases were found in regions with more population. Vasquez-Apestegui et al. [20] showed that in Lima (Peru), the rate of COVID-19 spread was associated with higher exposure to gaseous pollutants, and Andrée [21] concluded the same in his study on the Netherlands. Furthermore, exposure to air pollution could increase vulnerability and have detrimental effects on people affected by COVID-19 [22].

In addition, COVID-19 is spread by close human-to-human contact, so population density may be an important aspect in explaining spread [23,24], as people are more likely to be in close proximity [25]. Denser environments facilitate human interaction, which in turn can lead to higher infection rates [26].

There is no clear answer in the literature about whether population density can influence the number of COVID-19 cases. Some research claims that it is an explanatory factor for the spread of the virus, as concluded by Kadi and Khelfaoui [27] for Algeria, Kodera et al. [28] for Japan and Bhadra et al. [29] for India. However, some studies have concluded that population density is not associated with the spread of COVID-19, as explained by Fang and Wahba [30] for China or Hamidi et al. [31] for the United States, who report that counties with higher densities have lower COVID-19 mortality rates than counties with lower densities, due in part to better health care systems. Data for the population density variable are from the World Bank. All data used are the latest available and published.

## 2. Materials and Methods

COVID-19 cases may be associated with many factors, such as available health resources or health expenditure [32,33], policies adopted by countries to contain the pandemic [34,35] or compliance with hygiene and sanitary measures [36,37]. However, it may also be associated with environmental factors, which have not been sufficiently explored, especially in Europe; we aim to fill this gap in the literature. In this paper we analyse whether variables such as the emission of carbon monoxide gases (carbon), the pollutant emissions from transport (emissions), the population density (density), the average age of inhabitants (age) or restrictive policies have significant effects on the total number of cumulative COVID-19 cases per million inhabitants (cases) in Europe. The information on COVID-19 data refers to cumulative cases up to the beginning of July 2021, when the data collection took place.

**Figure 1:**
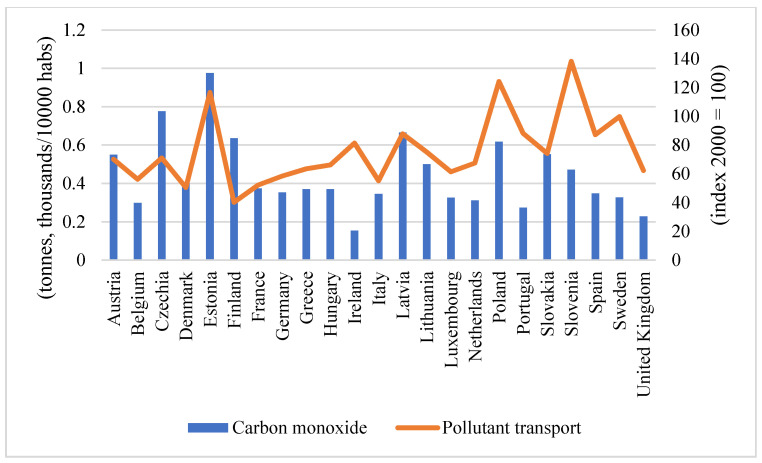
Pollutant emissions in European countries. Source: own elaboration.

In this work we have used as environmental variables, the emission of carbon monoxide (CO) pollutant gases per 10,000 inhabitants (tonnes, thousands), extracted from the OECD (The data can be downloaded from https://stats.oecd.org/Index.aspx?DataSetCode=MUNW, Accessed 1 July 2021) and pollutant emissions from transport, an indicator obtained from the Eurostat database (The data can be downloaded from https://ec.europa.eu/eurostat/databrowser/view/t2020_rk300/default/table?lang=en, Accessed 1 July 2021), because emissions from transport are the main contributor to air pollution. This indicator analyses the transport emissions of nitrogen oxides (NOx), non-methane volatile organic compounds (NMVOCs) and particulate matter (PM10). The indicator is an index to year 2000 (index 2000 = 100). The data for the European countries analysed in this work, for which all information is available, are shown in Figure 1.

Slovenia, Poland and Estonia were the countries with the highest levels of pollutant emissions from transport, while Finland and Denmark had the lowest levels. Estonia (again), Czechoslovakia and Latvia had the highest levels of carbon monoxide, and Ireland was the healthiest country in this respect, with the lowest level of carbon monoxide.

The age of the population (age) could also have an effect on COVID-19 chaos, as it is usually the younger population that is least affected [38], so regions with a younger population structure may have fewer cases. Therefore, the average age of the population in each country was used as a variable to determine whether the population structure influences the incidence of COVID-19 cases.

On the other hand, the restrictive policies imposed by the countries (policies), measured by the Government Response Stringency Index [39], was obtained from One World Data. This index shows the level of stringency or harshness of policies imposed by different governments but not their effectiveness. It is based on nine response indicators, including school closures or travel bans. This index can take a value from 0 to 100, where 100 means the policies imposed are more stringent. 

Several studies, such as those by Buja et al. [40] and Kenyon [41], propose OLS (ordinary least squares) regression models to analyse the effect of possible variables affecting the spread of COVID-19. Following this trail, to test the relationship among these variables, we have proposed the following multiple linear regression model:

$${\hat{Cases}}_{i}=\beta_{0\;}+\beta_{1\;\;}Carbon_{i}+\;\beta_{2}\;Pollutant_{i}+\beta_{3}\;Density_{i}+\beta_{4}\;Age_{i}+\beta_{5}\;Policies_{i}+\varepsilon_{i}$$

The number of cumulative coronavirus cases per million population varies between countries. The highest incidence per million population was in Czechia (155,837.9 cases/million population), which contrasts with Finland (17,142.8 cases/million population). The data shown are those reported up to the beginning of July 2021 and are taken from Johns Hopkins University. 

Population density was also dispersed. The Netherlands had the highest population density (421 km^2^/inhab) compared to Finland (18 km^2^/inhab), which had the lowest. The highest median age was in Germany and the lowest in Ireland. The highest value for the Government Response Stringency Index was in Italy and the lowest in Slovakia. The highest emission of carbon monoxide pollutants per 10,000 inhabitants was in Latvia and the lowest in Ireland. The mean was 0.44, and the standard deviation was 0.18. Slovenia had the highest pollutant emissions from transport indicator and Finland the lowest. The mean was 75.98 with a standard deviation of 24.01. Table 1 shows the main descriptive measures. Since there was considerable variability in some variables, the median and the interquartile range were calculated in addition to the mean. A total of 50% of the countries analysed had almost 80,000 coronavirus cases per 1,000,000 inhabitants.

## 3. Results

Before carrying out the regression analysis, it was checked that, in addition to non-multicollinearity, the hypotheses of independence, homoscedasticity and normality necessary to guarantee the validity of the model were verified. The errors, i.e., the differences between the observed values and those predicted by the model, were calculated. The Durbin–Watson statistic had a value of 2.29 so that the errors can be assumed to be independent. Furthermore, the assumptions of homoscedasticity and normality of the residuals were found to be fulfilled, since the correlation between the residual values in absolute values and the predicted scores was 0.19 and the Kolmogorov–Smirnov test for normality had a value of *p* = 0.94.

The results of the multiple regression analysis are shown in Table 2. The environmental variables studied have a positive effect on the incidence of COVID-19 cases. Both pollutant emissions from transport (at the 5% level) and emission of CO gases (at the 10% level) are associated with the incidence of COVID-19 cases. These results are significant and consistent and are in line with those presented by Ali and Islam [14], Lin et al. [13] and Frontera et al. [19] in relation to the emission of pollutant gases. These results show the importance of sustainability and the need to protect the environment to improve the health of citizens. An important factor that accentuates many diseases, such as COVID-19, is environmental pollution.

Population density has a positive influence. The higher the population density, the higher the number of cases per 1,000,000 inhabitants. These results are consistent with the information described by Arbel et al. [26] and in line with some research developed for other regions [28,29]. Although COVID-19 is most lethal to the adult population, no association has been found between those areas with a higher percentage of elderly population and the number of COVID-19 cases, although it is true that, in the case of contracting the disease, the chances of fatality among the elderly are higher [42].

On the other hand, the severity of the policies imposed by the countries was found to have influenced the number of cases of COVID-19, so, although they are necessary to contain the pandemic, they do not seem to be as decisive as other factors. 

## 4. Conclusions

The COVID-19 pandemic has caused millions of cases and deaths worldwide, largely affecting the European continent. Given the importance given to sustainability and the reduction in pollutant gases in recent years, the main objective of this study was to determine whether pollutant emissions are associated with an increased number of COVID-19 cases in Europe. Slovenia, Poland and Estonia were found to be the countries with the highest levels of pollutant emissions from transport, while Finland and Denmark had the lowest levels. Estonia, Czequia and Latvia had the highest carbon monoxide levels, and Ireland was the country with the lowest carbon monoxide levels.

The results obtained indicate that the emission of carbon monoxide pollutant gases and pollutant emissions from transport positively affect the incidence of COVID-19. Other demographic variables that could have had an impact on the number of coronavirus cases were also included. There is no evidence that countries with a higher proportion of adult population have more cases of COVID-19. However, there was a positive association between population density and the number of cases, with countries with higher density being more affected, partly because COVID-19 is spread by close contact between people, and higher density may lead to a higher risk of contact. Greater severity or toughness on the part of governments in taking measures to contain the pandemic has not been a determining factor in the number of COVID-19 cases. The results show the importance of sustainability and the need to protect the environment to improve the health of citizens. 

To mitigate the effect of COVID-19, in addition to monitoring compliance with basic measures, efforts should be made to reduce environmental contamination. Several studies have shown the association of polluted gases with COVID-19 cases, and this study has also shown this to be true for the European continent. This is why not only should the latest developments already enacted by the European Commission with the adoption of the 2030 Agenda and the 17 Sustainable Development Goals be followed, but tougher sanctions and measures should be imposed when pollution thresholds are exceeded. In addition, companies should be taxed more heavily for emitting polluting gases and should even be penalised in order to regulate and control these emissions. Governments should also provide incentives to the population to buy fully sustainable products, such as electric cars or solar panels, in order to contribute to the reduction in polluting gases. The results are representative at the country level, although the particularities of each region should be taken into account when making decisions, although the aim of this work was to provide a global vision of environmental contamination and its possible influence on the COVID-19 cases.

One of the limitations of this study is that it is limited in time, as the data is changing, and the accumulated cases of COVID-19 used in this study are only those reported up to the beginning of July 2021. For future research, it would be advisable to repeat this study by including more variables that could affect the spread of COVID-19 or by breaking it down in detail by the different types of pollutant gases. 

As a future line of research, carrying out this study considering the main European cities and each country separately is recommended to see if there are differences with respect to this research for the countries at a global level.

## Figures and Tables

**Table 1:** Main descriptive measures.

Variables	Minimum	Maximum	Mean	Standard Deviation	Median	Interquartile Range
Cases per 1,000,000 inhab.	17,142.8	155,837.9	79,955.9	30,267.9	79,980.24	28,362.5
Carbon (tonnes, thousands/10,000 habs)	0.1545	0.9755	0.4446	0.1872	0.3702	0.2266
Emissions (index 2000 = 100).	40.2	138.2	75.98	24.01	70.10	29.30
Population density (Km^2^/inhab)	18.2	421	124.16	98.65	106.7	95.16
Age	37.8	47.8	43.07	2.19	43.30	2.80
Policies (0–100)	46.3	87.9	67.89	11.66	69.44	22.22

The highest variance inflation factor is equal to 1.75, which shows the absence of multicollinearity problems.

**Table 2:** Multiple regression model.

Variables	Unstandardised Coefficients	Standardised Coefficients	*p*-Value
(Constant)	94,465.6 (113,142.7)		0.41
Carbon (tonnes, thousands/10,000 habs)	72,831.1 * (39,135.4)	0.45	0.08
Emissions (index 2000 = 100).	572.3 ** (252.7)	0.45	0.03
Population density (Km2/inhab)	131.0 ** (60.0)	0.46	0.04
Age	−4042.5 (2932.3)	−0.29	0.18
Policies (0–100)	957.9 (568.2)	0.37	0.11

Standard error in parentheses ** Significant at 5% level. * Significant at the 10% level.

## Data Availability

Not applicable.
